# Increased Hydrostatic Pressure Promotes Primary M1 Reaction and Secondary M2 Polarization in Macrophages

**DOI:** 10.3389/fimmu.2020.573955

**Published:** 2020-10-14

**Authors:** Bo Wang, Maren Kasper, Björn Laffer, Gerd Meyer zu Hörste, Susanne Wasmuth, Martin Busch, Tida Viola Jalilvand, Solon Thanos, Arnd Heiligenhaus, Dirk Bauer, Carsten Heinz

**Affiliations:** ^1^Ophthalmology and Ophtha-Lab at St. Franziskus Hospital, Münster, Germany; ^2^Institution of Neurology and Institution for Translational Neurology, Universitätsklinikum Münster, Münster, Germany; ^3^Institution of Experimental Ophthalmology, Westfälische Wilhelms-Universität, Münster, Germany; ^4^Ophthalmology, University of Duisburg-Essen, Essen, Germany

**Keywords:** macrophage, increased hydrostatic pressure, secondary glaucoma, dexamethasone (DEX), ECM-extracellular matrix, M1/M2 balance

## Abstract

Patients with chronic anterior uveitis are at particularly high risk of developing secondary glaucoma when corticosteroids [e.g., dexamethasone (Dex)] are used or when inflammatory activity has regressed. Macrophage migration into the eye increases when secondary glaucoma develops and may play an important role in the development of secondary glaucoma. Our aim was to evaluate *in vitro* if increased hydrostatic pressure and corticosteroids could induce changes in macrophages phenotype. By using a pressure chamber cell culture system, we assessed the effect of increased hydrostatic pressure (HP), inflammation, and immunosuppression (Dex) on the M1/M2 phenotype of macrophages. Bone marrow-derived macrophages (BMDMs) were stimulated with medium, lipopolysaccharide (LPS, 100 ng/ml), Dex (200 ng/ml), or LPS + Dex and incubated with different HP (0, 20, or 60 mmHg) for 2 or 7 days. The numbers of CD86+/CD206− (M1 phenotype), CD86–/CD206+ (M2 phenotype), CD86+/CD206+ (intermediate phenotype), F4/80+/TNF-α+, and F4/80+/IL-10+ macrophages were determined by flow cytometry. TNF-α and IL-10 levels in cell culture supernatants were quantified by ELISA. TNF-α, IL-10, fibronectin, and collagen IV expression in BMDMs were detected by immunofluorescence microscopy. Higher HP polarizes macrophages primarily to an M1 phenotype (LPS, 60 vs. 0 mmHg, d2: *p* = 0.0034) with less extra cellular matrix (ECM) production and secondary to an M2 phenotype (medium, 60 vs. 0 mmHg, d7: *p* = 0.0089) (medium, 60 vs. 20 mmHg, d7: *p* = 0.0433) with enhanced ECM production. Dex induces an M2 phenotype (Dex, medium vs. Dex, d2: *p* < 0.0001; d7: *p* < 0.0001) with more ECM production. Higher HP further increased M2 polarization of Dex-treated macrophages (Dex, 60 vs. 0 mmHg, d2: *p* = 0.0417; d7: *p* = 0.0454). These changes in the M1/M2 phenotype by high HP or Dex treatment may play a role in the pathogenesis of secondary uveitic glaucoma- or glucocorticoid (GC)-induced glaucoma.

## Introduction

Glaucoma represents a group of chronic optic neurodegenerative diseases and is one of the leading causes of blindness worldwide. Although family history, age, and ethnicity may have an impact on glaucoma, the most important risk factor known so far is elevated intraocular pressure (IOP) (1). Glaucoma is characterized by optic nerve head damage, loss of optic nerve axons, and retinal ganglion cell death (2). Glaucoma can be further divided into primary and secondary types. Reducing IOP is the only proven way to treat or slow the progression of glaucoma (3).

Uveitis is a group of ocular inflammatory diseases that primarily involve uveal tissues. Uveitis can develop at all ages and in all races and often occurs in young and middle-aged adults. Uveitis frequently takes a recurrent course, ultimately leading to serious complications, such as secondary cataract, glaucoma, macular edema, retinal scars, and optic neuropathy, and resulting in visual impairment and blindness. Reducing inflammation by treating the eye with corticosteroids [e.g., dexamethasone (Dex)] constitutes the first-line therapy for uveitis. However, corticosteroids put patients at increased risk for undesired side effects, as these drugs may increase uveitis patients' risk of developing secondary cataract, elevated IOP, and secondary glaucoma (4).

Often, IOP first increases when inflammatory activity has been eliminated, possibly due to reduced uveoscleral outflow (5). It is generally believed that secondary open-angle glaucoma develops as a result of chronic changes in the trabecular meshwork (TM) outflow pathway.

Glucocorticoids (GCs) may modify gene expression in TM cells and tissues, as shown for myocilin and fibronectin (6, 7). As a result, the aqueous outflow resistance increases and thereby elevates the IOP, which can also be found in primary open-angle glaucoma patients.

Macrophages are widely distributed in various tissues and organs, including the eye. Recent studies showed that neuroinflammation is closely related to the pathogenesis of glaucoma (8), and macrophages/monocytes play an important role in regulating outflow and IOP (9, 10). One previous study showed that the number of macrophages increased in the TM of rabbit eyes after selective laser trabeculoplasty, and infusing macrophages into the anterior chamber increased AH outflow from 24 h to 4 days (11).

Macrophages comprise a group of cells with strong plasticity, heterogeneity, and immunological functions, which are closely related to various physiological and pathological processes. They can be divided into classically activated (M1, proinflammatory functions) or alternatively activated (M2, repair or regulatory functions) macrophages (12, 13).

Moreover, various cytokines related to macrophages (e.g., TNF-α, TGF-β, IL-1β, IL-6, IL-8, IL-10, and IL-12) are known to affect outflow and neuroinflammation in glaucoma (14). Various experimental studies have shown that macrophages infiltrate the eye and the retina when glaucoma develops and that depleting macrophages by intravitreally administering clodronate liposomes reduces retinal ganglion cell loss, which suggests that macrophages have a detrimental effect in this setting (15).

In pathologic situations, macrophages are found to be key components of tissue repair and remodeling that occur during wound healing, allergy, parasite infection, and cancer. They may also interact with stem or progenitor cells, which are further components in repair and remodeling (16). For example, a previous study has demonstrated that a circulating cell population with both M1 and M2 macrophage surface markers could characterize patients with systemic sclerosis with lung involvement. These macrophages are also associated with systemic sclerosis related interstitial lung disease, higher systolic pulmonary artery pressure, and antibodies against anti-topoisomerase (a predictor for lung involvement) (17).

A similar situation could be found in patients with chronic anterior uveitis, who are at particular high risk of developing secondary glaucoma, even when corticosteroids are used or when inflammatory activity has regressed. We hypothesized that changes in the HP might be associated with disparate macrophage effector functions. We questioned if increased hydrostatic pressure and corticosteroids could induce changes in the macrophage phenotype. In this study, therefore, we used a pressure chamber cell culture system to determine the M1/M2 phenotype of macrophages under increased hydrostatic pressure (HP) to increase our knowledge of the processes macrophages might undergo during glaucoma development and progression.

## Materials and Methods

### Mice

Female C57/BL6J mice (6–8 weeks) were purchased from Charles River Wiga (Sulzfeld, Germany) and used to isolate macrophages for all experiments. Previous secondary glaucoma studies have shown no statistically significant differences in gender with respect to the expression of cytokines, chemokines or MMPs in the AH (18). The protocol was approved by the North Rhine-Westphalia State Agency for Nature, Environment, and Consumer Protection (LANUV) (AZ 81-02.05.50.19.006). The use of animals was in accordance with the Institutional Animals Care and Use and Ethics Committee, and with the ARVO Statement for the Use of Animals in Ophthalmic and Vision Research.

### Bone Marrow-Derived Macrophages

Bone marrow-derived macrophages (BMDMs) were obtained from two femurs of female C57/Bl6 mice and 2 × 10^6^ nucleated cells were cultured in 10 ml RPMI 1640 medium containing 10% fetal calf serum and 15% L929 cell-conditioned media in 10-cm untreated tissue culture dishes (VWR, Germany). The conditioned medium was prepared of 5-day supernatant from L929 cell line (19–21).

After 7 days in culture, nonadherent cells were removed and adherent cells were harvested for assays using Accutase (Sigma-Aldrich, Taufkirchen, Germany). 1 × 10^6^ adherent cells in 1 mL RPMI 1640 medium containing 10% fetal calf serum were cultured in 6-well plates.

BMDMs with medium, 100 ng/mL LPS (eBioscience, Dreieich, Germany), 200 ng/mL Dex (Sigma-Aldrich, Taufkirchen, Germany), or LPS+Dex were cultured under different HPs for 2 or 7 days. Then, the cells were used for MTT testing, immunofluorescence staining, and flow cytometry. The cell culture supernatants were harvested and used for ELISA (22).

The connection of Toll like receptor-4 (TLR-4) and its ligand lipopolysaccharide (LPS) with IRI or glaucoma has been demonstrated in previous studies, showing that genetic deletion of TLR-4 is neuroprotective in IRI disorders (23). Furthermore, mutations on the TLR-4 correlated with altered susceptibility to primary open angle glaucoma and were even described as a possible bio-indicator (24).

### Chamber System for Hydrostatic Pressure

The pressure chamber system has been described previously (25). In brief, cell culture plates were positioned in the pressure chambers consisting of rust-free steel, 15 cm in diameter and 10 cm in depth. Each chamber has an external connector for adjusting pressure with an external pressure meter. The chamber system can be used to reproducibly establish normal or increased HP conditions for cell culture and is able to maintain constant pressures for several days. Here, 0 mmHg (room pressure) was set as control, 20 mmHg was set to mimic normal IOP, and 60 mmHg was set to mimic increased IOP. BMDMs were cultured under different HP for 2 or 7 days. The pressures in the chamber were checked 4 times per day.

### MTT Conversion Assay

To determine the effect of increased HP on the viability of BMDMs, cells (1 × 10^6^ BMDMs/mL) were treated with medium, LPS, Dex, or LPS + Dex under various HP for 2 or 7 days. For the experiments, 3-(4,5-dimethylthiazol-2-yl)-2,5-diphenyltetrazolium bromide (MTT, Carl Roth, Karlsruhe, Germany) solution prepared by dissolving MTT (5 mg/mL) in RPMI 1640 was added and incubated for 3 h at room pressure. Afterwards, cells and dye crystals were dissolved by adding *N, N*-dimethylformamide. Absorbance was measured at 570 nm (reference at 690 nm) in an ELISA reader (MRX-ELISA; Dynatech Laboratories, Sussex, UK). The results were expressed as optical density (OD) (26).

### Antibodies

Antibodies for immunofluorescence were purchased from the following sources: F4/80 (0.2 μg/ml, BM8, OriGene, Rockville, MD, USA), TNF-α (2.5 μg/ml, MP6-XT3, eBioscience, San Diego, CA, USA), IL-10 (2.5 μg/ml, JES5-2A5, eBioscience), fibronectin (0.7 μg/ml, Polyclonal, invitrogen, Carlsbad, CA, USA), and collagen IV (2.5 μg/ml, Polyclonal, Invitrogen). Secondary antibody were goat anti-rat IgG Alexa Fluor® 488 (2 μg/ml, BioLegend, San Diego, CA, USA), streptavidin Alexa Fluor® 594 (0.5 μg/ml, BioLegend), goat anti-rabbit IgG Alexa Fluor® 488 (2 μg/ml, Invitrogen).

The following antibodies (BioLegend) were used for flow cytometry: APC anti-mouse F4/80 (2.5 μg/ml, BM8), PE/Cy7 anti-mouse CD86 (1.25 μg/ml, GL-1), PE anti-mouse CD206 (1.25 μg/ml, C068C2), PE/Cy7 anti-mouse TNF-α (5 μg/ml, MP6-XT2), and Alexa Fluor® 488 anti-mouse IL-10 (12.5 μg/ml, JES5-16E3).

### Immunofluorescence Staining

BMDMs, cultured on 8-chamber slides (Millicell® EZ SLIDES, MerckKGaA, Darmstadt, Germany) with different HP and treatments, were fixed in 4% paraformaldehyde (Carl Roth, Karlsruhe, Germany) for 30 min and blocked with 5% goat serum in 1x Perm Buffer (eBioscience) for 1 h at room temperature. Cells were then incubated with the primary antibody overnight at 4°C. After washing with PBS, the secondary antibody was added for 30 min. Cells were counterstained with Hoechst at 1:1,000 (B-2261; Sigma-Aldrich, St. Louis, Missouri, USA) for 10 min at room temperature before embedding with moviol (Sigma-Aldrich) (27). The stainings were analyzed with a confocal microscope (LSM 710; Carl Zeiss, Jena, Germany) and images were acquired by ZEN acquisition software (2012; Carl Zeiss). For each marker, nine pictures from three independent experiments were randomly taken. The mean fluorescence intensity (MFI) and number of cells (based on nuclei within the viewing/visual field/field of vision) were quantified using ImageJ (1.80, National Institutes of Health, USA) (28, 29).

### Flow Cytometry Analysis of BMDMs

BMDMs were cultured under different HPs and types of stimulation for 2 or 7 days. For flow cytometric analysis, cells were treated with Brefeldin A (eBioscience) for 6 h and subsequently removed from culture dishes using Accutase (Biowest, Nuaille, France). Cells were then blocked with Fc block (2 μl/10^6^ cells, BD Biosciences, Hamburg, Germany) for 5 min at 4°C to avoid unspecific staining. To characterize macrophages, cells were stained using antibodies targeting F4/80, CD86, CD206, TNF-α, and IL-10 (27). Data were collected with a flow cytometer CytoFLEX (Beckman Coulter GmbH, Krefeld, Germany), and Kaluza Analysis software 2.1. (Beckman Coulter GmbH) was used for analysis.

### Cytokine Quantification by ELISA

Supernatants from BMDMs with or without stimulation were harvested 2 or 7 days after incubation with different HPs and then stored at −20°C. In the supernatants, the content of TNF-α or IL-10 (BioLegend, Koblenz, Germany) was determined by ELISA (27, 30).

### Statistical Analysis

Data were analyzed using Graph Pad Prism software version 7 (La Jolla, CA, USA). All data were analyzed using the Kolmogorov-Smirnov and Shapiro-Wilk tests to check the normal distribution of the data. A normal distribution was found in all subgroups. To determine the effects of treatment (medium, LPS, Dex, or LPS+Dex) and the level of hydrostatic pressure (0, 20, or 60 mmHg), two-way analysis of variance (ANOVA) followed by Tukey's multiple comparisons test was used to determine significant differences.

Results were shown as mean ± standard deviation (SD). *P* < 0.05 was considered statistically significant. All experiments were repeated independently at least three times. The figures were generated using Graph Pad Prism 7.

## Results

### Influence of Increased Hydrostatic Pressure on the Viability of BMDMs

**Figure 1:**
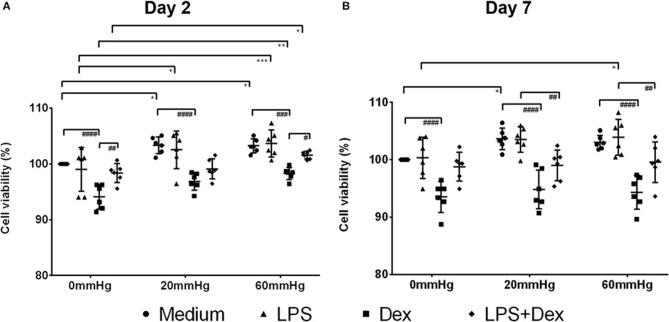
MTT conversion assay of BMDMs under different hydrostatic pressures. MTT conversion (%) (as a marker for cell viability) of medium, LPS, Dex, or LPS + Dex-treated BMDMs at 0, 20, or 60 mmHg on day 2 **(A)** and day 7 **(B)** (*n* = 6 independent experiments). The OD value in the medium group at 0 mmHg served as control and the viability was set as 100%. Statistical significance between different pressures: *p < 0.05, **p < 0.01, ***p < 0.001; Statistical significance between different treatments: ^#^*p* < 0.05, ^*##*^*p* < 0.01, ^*###*^*p* < 0.001, ^*####*^*p* < 0.0001. Data were analyzed by two-way ANOVA and Tukey's *post hoc* test.

Initially, MTT conversion was measured to evaluate the impact of increased HP on the viability of BMDMs. Stimulating BMDMs for 2 days with LPS did not result in any significant viability changes compared to medium control (Figure 1A). However, MTT conversion increased significantly when HP was higher (60 or 20 mmHg) than room pressure (0 mmHg) (*p* < 0.05). A significant decrease in MTT conversion was measured after Dex treatment in general as compared to Med (*p* < 0.0001) but Dex and LPS combined increased MTT conversion in a culture of BMDMs as compared to Dex alone (Figure 1A). After culture for 7 days, still no significant differences in viability were found between medium and LPS groups under the same HP (Figure 1B). With LPS treatment MTT conversion increased with higher pressure (60 mmHg) as compared to 0 mmHg (*p* = 0.0304). Significantly decreased MTT conversion was found in the Dex-treated group compared to medium control (*p* < 0.0001) (Figure 1B).

### Influence of Increased Hydrostatic Pressure on Macrophage Phenotype

The phenotype of adherent bone marrow cells was then analyzed by flow cytometry in an introductory experiment. Adherent bone marrow-derived cells were stained with an antibody targeting the murine F4/80 antigen and Fix Viability Dye eFluor 780 as a marker for cell viability. Here, 92.01 ± 4.25% of all single cell events were viable and, of all viable cells, 93.46 ± 3.17% were F4/80 positive, indicating that they belong to the macrophage lineage (Supplementary Figure 1).

**Figure 2:**
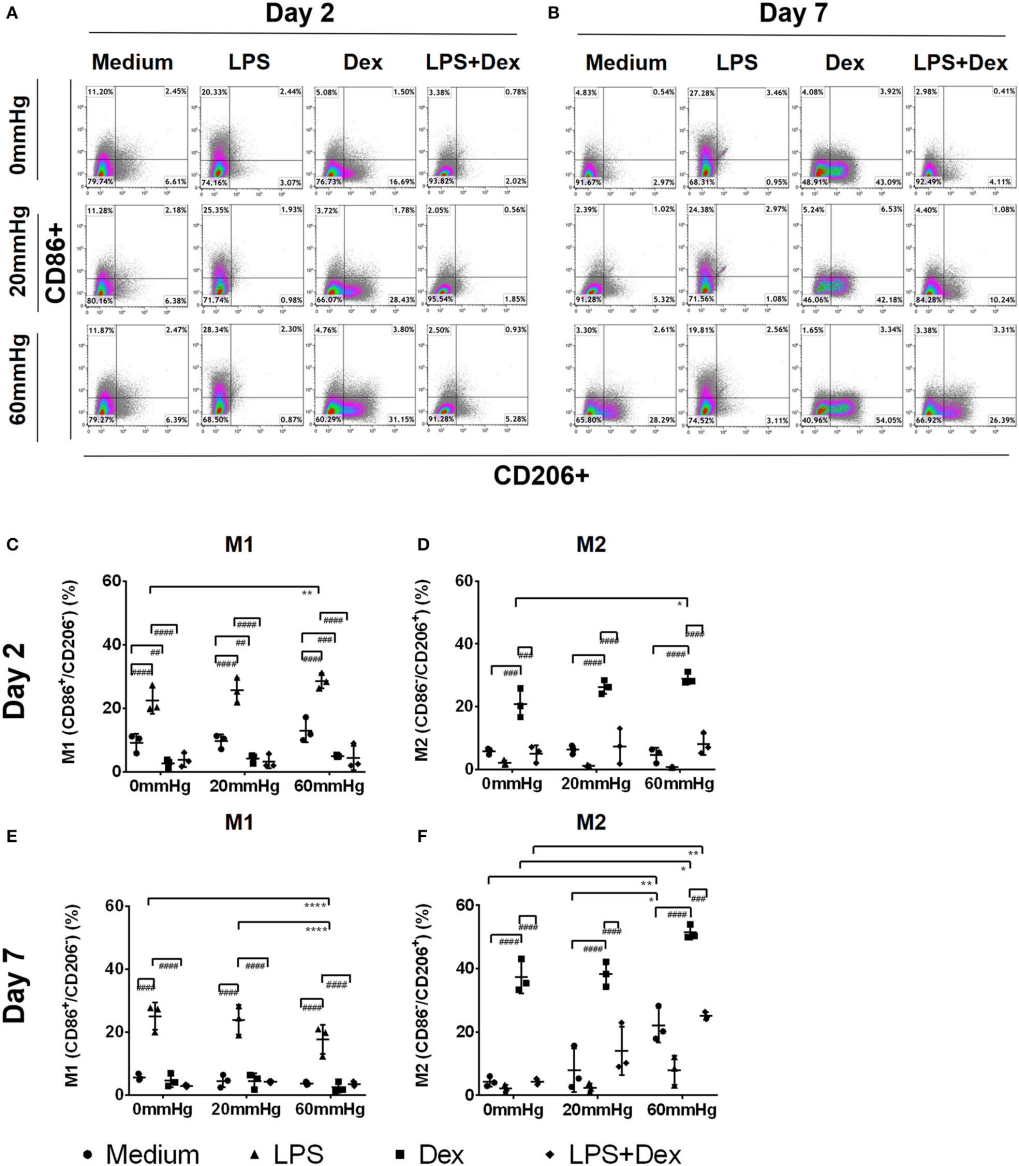
Frequency of M1, M2, or M1/2 subpopulations in BMDMs under increased HP. To calculate differences in the M1/M2 populations, BMDMs were stained using antibodies targeting F4/80, CD86, and CD206 and the specimens were analyzed by flow cytometry. Dotplots showing CD86+/CD206– (upper left area), CD86–/CD206+ (lower right area), or CD86+/CD206+ (upper right area) populations on day 2 **(A)** and day 7 **(B)** by flow cytometry. Graphs showing the calculated frequency of CD86+/CD206– **(C)**, or CD86–/CD206+ **(D)**, events on day 2 (*n* = 3). Graphs showing the calculated frequency of each group on day 7 **(E,F)** (*n* = 3). Statistical significance between different pressures: *p < 0.05, **p < 0.01, ****p < 0.0001; Statistical significance between different treatments within one pressure: ^*##*^*p* < 0.01, ^*###*^*p* < 0.001, ^*####*^*p* < 0.0001. Data were analyzed by two-way ANOVA and Tukey's *post hoc* test.

The 2-day culture under increased HP initiated a proinflammatory (M1) phenotype in macrophages, as indicated by the increased frequency of CD86+/CD206– events (M1) in BMDMs with LPS (Figures 2A,C). In the LPS group, the frequency of M1 macrophages at 60 mmHg was significantly higher than 0 mmHg (*p* = 0.0034) (Figures 2A,C). In contrast, 2-day culture under Dex treatment skewed the BMDMs to an anti-inflammatory phenotype (M2), as indicated by the increased frequency of CD86–/CD206+ events (Figures 2A,D). Further incubation of BMDMs with 60 mmHg HP significantly enhanced this effect, as reflected by the increased frequency of M2 macrophages as compared to 0 mmHg (*p* = 0.0417) (Figures 2A,D).

After stimulating BMDMs with LPS and Dex, upregulation of M1 frequency was significantly weaker than after LPS alone (*p* < 0.0001) (Figure 2C), while the frequency of M2 events was lower than after Dex alone (*p* < 0.001) (Figure 2D). The frequency of the intermedium phenotype (CD86+/CD206+) was low in each group on day 2, and no significant difference was found between them (data not shown).

Seven-day culture under increased HP resulted in a pronounced anti-inflammatory phenotype of macrophages, as indicated by a lower frequency of M1 but higher frequency of M2 macrophages with or, even more pronounced, without LPS (Figures 2B–F). In medium controls, the M2 frequency in the 60 mmHg group was significantly higher than in both the 0 mmHg (*p* = 0.0089) and 20 mmHg groups (*p* = 0.0433) (Figures 2B,F). With LPS treatment, the differences in the frequency of M1 macrophages between 0 and 60 mmHg (*p* < 0.0001), or 20 and 60 mmHg (*p* < 0.0001), both reached the level of significance (Figures 2B,E).

Seven days of culture under treatment with Dex resulted in a more pronounced anti-inflammatory phenotype distortion of BMDM compared to day 2 (Figures 2B,D), as demonstrated by further upregulation of M2 frequency after culture with Dex (Figures 2B,F). Increased HP enhanced this upregulation and the highest M2 frequency was found when Dex-treated cells were cultured at 60 mmHg which was significantly higher than at 0 mmHg (*p* = 0.0454) (Figures 2B,F). After stimulating BMDMs with Dex in combination with LPS, the frequency of M2 macrophages was lower than for Dex alone. With LPS + Dex treatment, M2 frequency in the 60 mmHg group was significantly higher than in the 0 mmHg group (*p* = 0.0024) (Figures 2B,F).

### Influence of Increased Hydrostatic Pressure on the Expression of TNF-α and IL-10

Using the pressure chamber cell culture system, we analyzed the expression and release of TNF-α and IL-10 from BMDMs cultured under increased HP using immunofluorescence microscopy, flow cytometry, and ELISA.

### Production of TNF-α

**Figure 3:**
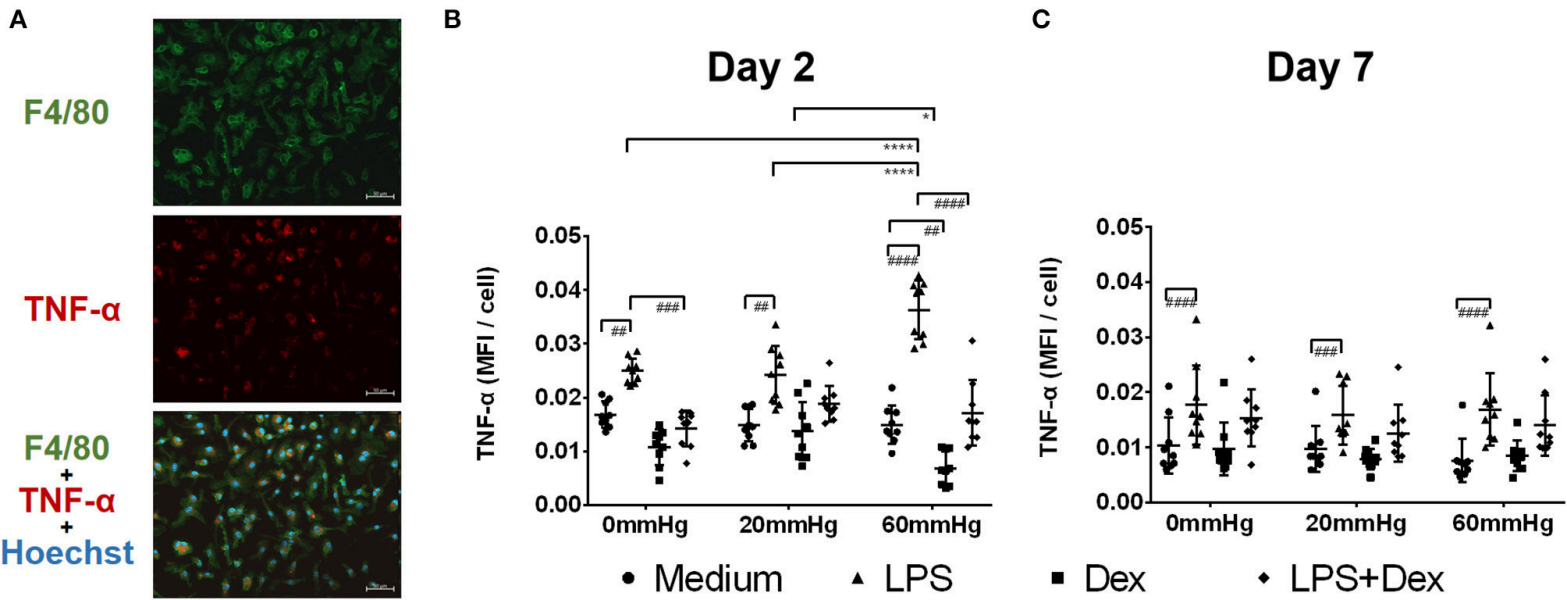
TNF-α expression in BMDMs under different hydrostatic pressures. **(A)** Representative immunofluorescence images of TNF-α (red) staining in BMDMs under different hydrostatic pressures. F4/80-positive (green) cells represent macrophages. Scale bar = 50 μm. **(B)** Graphs displaying the calculated MFI/cell of TNF-α on day 2 **(B)** and day 7 **(C)** (*n* = 9). Statistical significance between different pressures: *p < 0.05, ****p < 0.0001; Statistical significance between different treatments within one pressure: ^#^*p* < 0.05, ^*##*^*p* < 0.01, ^*###*^*p* < 0.001, ^*####*^*p* < 0.0001. Data were analyzed by two-way ANOVA and Tukey's *post hoc* test.

**Figure 4:**
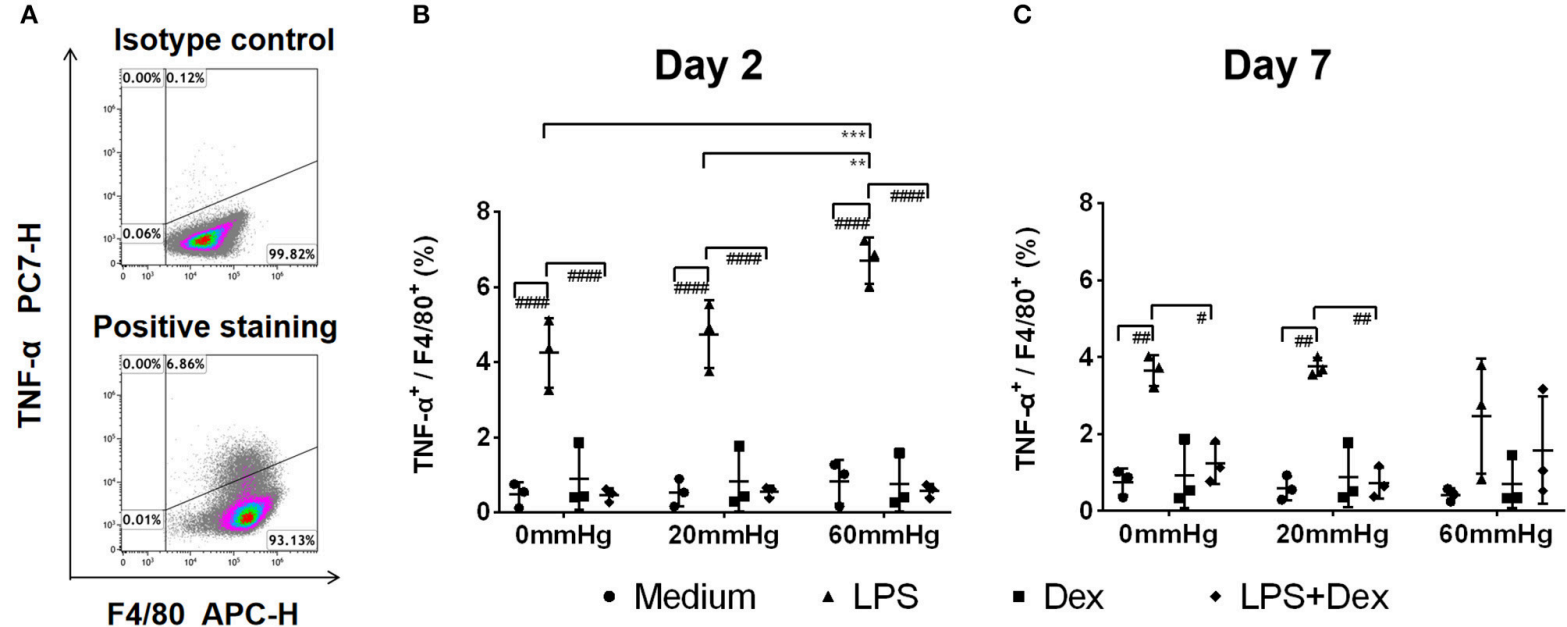
Frequency of F4/80^+^/TNF-α^+^ events under increased HP. To calculate differences in the F4/80+/TNF-α+ populations, BMDMs were stained with antibodies targeting F4/80 and TNF-α and were analyzed by flow cytometry. **(A)** Dotplots showing isotype control and positive staining of TNF-α in cells. Graphs showing the calculated frequency of F4/80^+^/TNF-α^+^ events on day 2 **(B)** and day 7 **(C)** (*n* = 3). Statistical significance between different pressures: **p < 0.01, ***p < 0.001; Statistical significance between different treatments within one pressure: ^#^*p* < 0.05, ^*##*^*p* < 0.01, ^*###*^*p* < 0.001, ^*####*^*p* < 0.0001. Data were analyzed by two-way ANOVA and Tukey's *post hoc* test.

**Figure 5:**
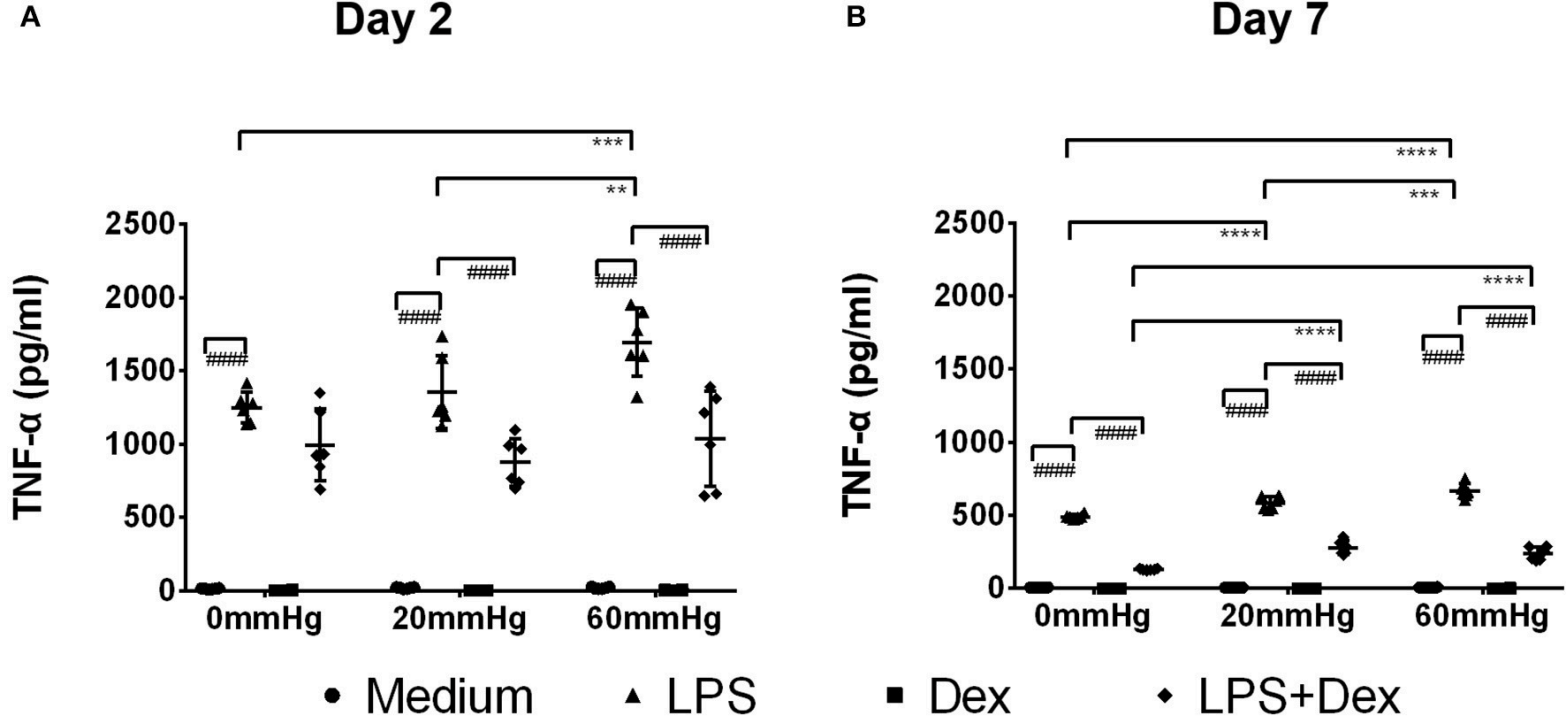
TNF-α levels in the supernatant of BMDMs under different hydrostatic pressures. Graphs displaying the calculated TNF-α levels in the supernatant from BMDMs on day 2 **(A)** and day 7 **(B)** (*n* = 6) as measured by ELISA. Statistical significance between different pressures: **p < 0.01, ***p < 0.001, ****p < 0.0001; Statistical significance between different treatments within one pressure: ^*####*^*p* < 0.0001. Data were analyzed by two-way ANOVA and Tukey's *post hoc* test.

The expression of TNF-α at 60 mmHg was significantly higher in LPS-stimulated cells after 48 h than at 0 mmHg (*p* < 0.0001) and 20 mmHg (*p* < 0.0001), as observed by immunofluorescence microscopy (Figures 3A,B). With LPS stimulation, the frequency of TNF-α+/F4/80+ events at 60 mmHg was significantly higher than at 0 mmHg (*p* = 0.0007) and 20 mmHg (*p* = 0.0052), as measured by flow cytometry (Figures 4A,B). On day 2 of culture under increased HP, BMDMs initiate increased production of TNF-α after activation with LPS, as measured in BMDM supernatants by ELISA (Figure 5A). The differences in TNF-α levels between the 0 mmHg and 60 mmHg groups (*p* < 0.0001), or the 20 and 60 mmHg groups (*p* = 0.0051), reached the level of significance (Figure 5A).

Dex treatment decreased the expression of TNF-α under increased pressure (60 mmHg) compared to 20 mmHg (*p* = 0.0470) as measured by immunofluorescence microscopy (Figure 3B). In the LPS + Dex group, expression of TNF-α was weaker than in the LPS group at each pressure level (*p* < 0.0001), as measured by fluorescence microscopy (Figure 3B). Significantly lower TNF-α levels were detected in the supernatant from the LPS + Dex group than from the LPS group (*p* < 0.0001) by using ELISA (Figure 5A).

After prolonged culture for 7 days, significantly lower levels of TNF-α could be found than after 2 days at the same HP level (Figure 3C). However, the differences in TNF-α expression between various pressures did not reach the level of significance in the medium or LPS groups as determined by immunofluorescence staining and flow cytometry (Figures 3C, 4C).

On day 7, the TNF-α level in the supernatant of BMDMs with LPS treatment cultured at 60 mmHg was significantly higher than at 0 mmHg (*p* < 0.0001) or 20 mmHg (*p* = 0.0006) as measured by ELISA. Furthermore, the TNF-α level at 20 mmHg was also significantly higher than in the 0 mmHg group (*p* < 0.0001) (Figure 5B).

In the Dex group, after 7 days, TNF-α expression in BMDMs (Figures 3C, 4C) and the TNF-α level in the supernatant (Figure 5B) were low and no significant difference could be found between different HPs. In the LPS + Dex group, a similarly increased TNF-α level with increased HP was found on day 7, and the differences between 0 and 20 mmHg (*p* < 0.0001), and 0 and 60 mmHg (*p* < 0.0001), both reached the level of significance (Figure 5B).

### Production of IL-10

**Figure 6:**
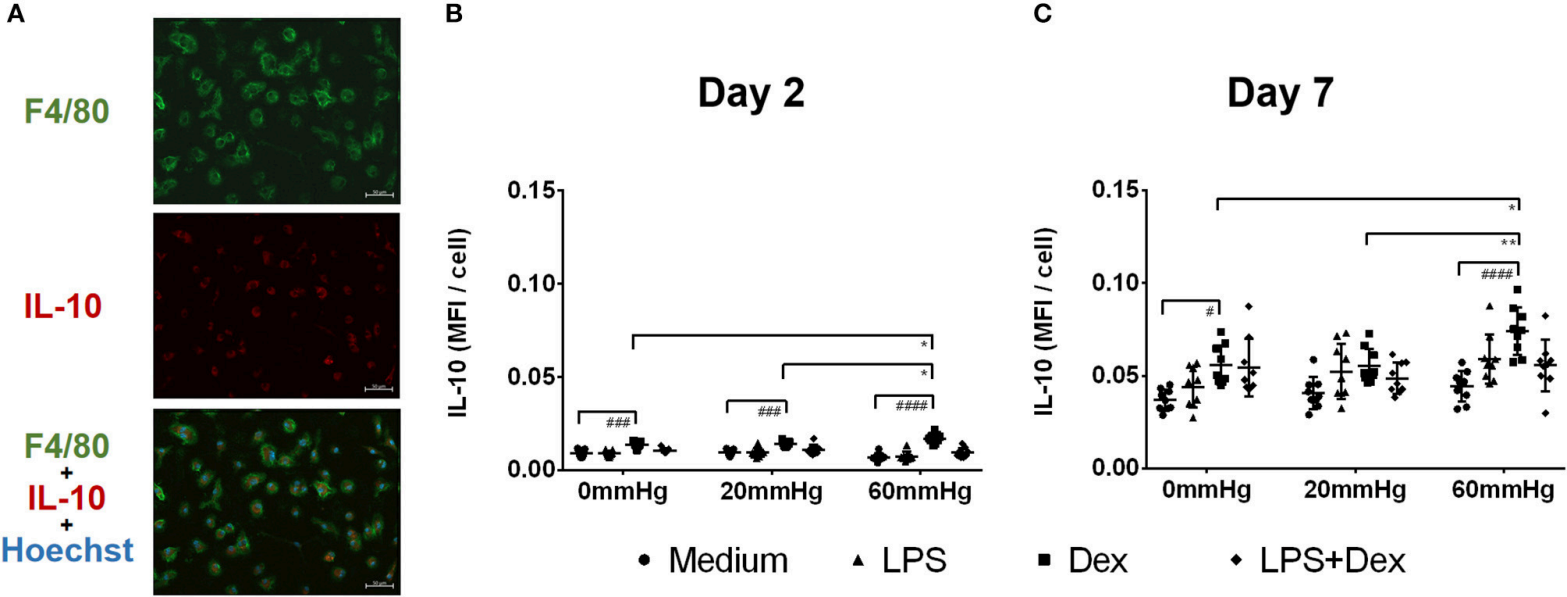
IL-10 expression in BMDMs under different hydrostatic pressures. **(A)** Representative immunofluorescence images of IL-10 (red) staining in BMDMs under different hydrostatic pressures. F4/80-positive (green) cells represent macrophages. Scale bar = 50 μm. Graphs displaying the calculated MFI/cell of IL-10 on day 2 **(B)** and day 7 **(C)** (*n* = 9). Statistical significance between different pressures: *p < 0.05, **p < 0.01; Statistical significance between different treatments within one pressure: ^#^*p* < 0.05, ^*###*^*p* < 0.001, ^*####*^*p* < 0.0001. Data were analyzed by two-way ANOVA and Tukey's *post hoc* test.

**Figure 7:**
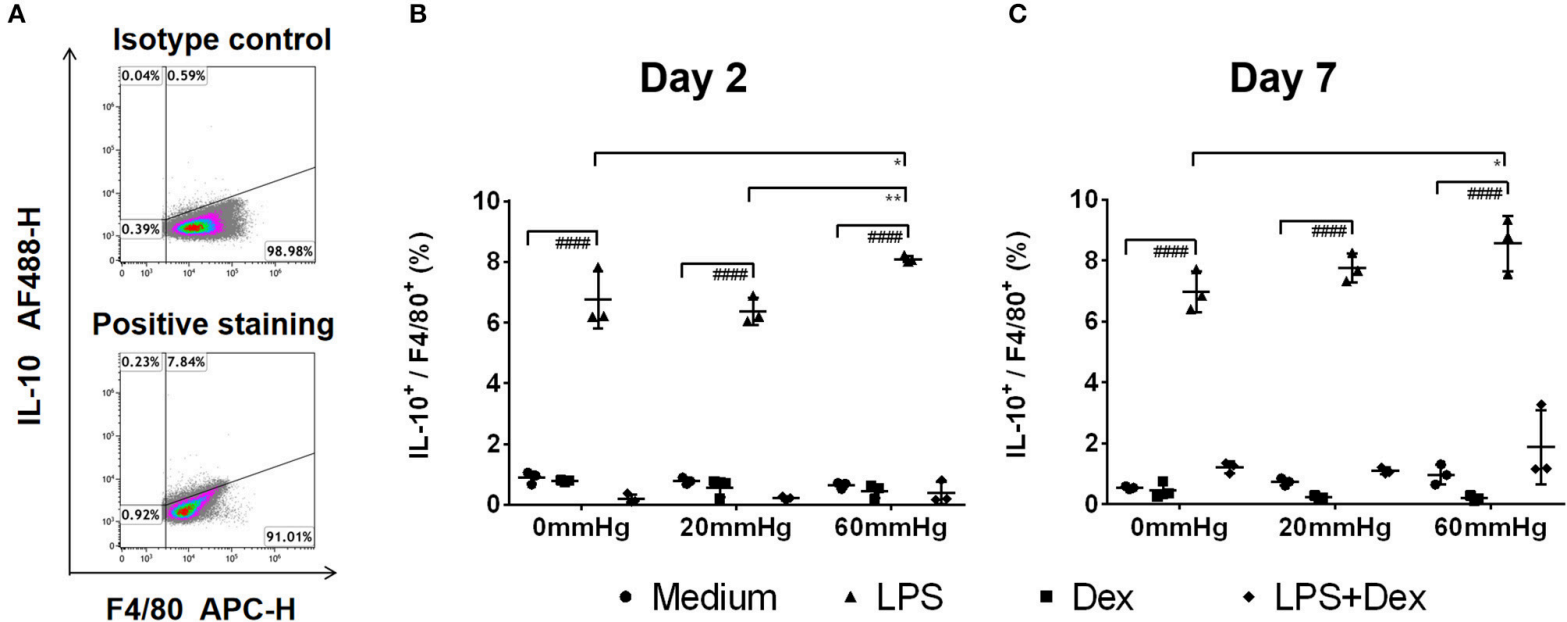
Frequency of IL-10^+^/F4/80^+^ events under increased HP. To calculate differences in the IL-10^+^/F4/80^+^ populations, BMDMs were stained with antibodies targeting F4/80 and IL-10. The specimens were then analyzed by flow cytometry. **(A)** Dotplots showing isotype control and positive staining of IL-10 in cells. Graphs showing the calculated frequency of IL-10^+^/F4/80^+^ events on day 2 **(B)** and day 7 **(C)** (*n* = 3). Statistical significance between different pressures: *p < 0.05, **p < 0.01; Statistical significance between different treatments within one pressure: ^*####*^*p* < 0.0001. Data were analyzed by two-way ANOVA and Tukey's *post hoc* test.

**Figure 8:**
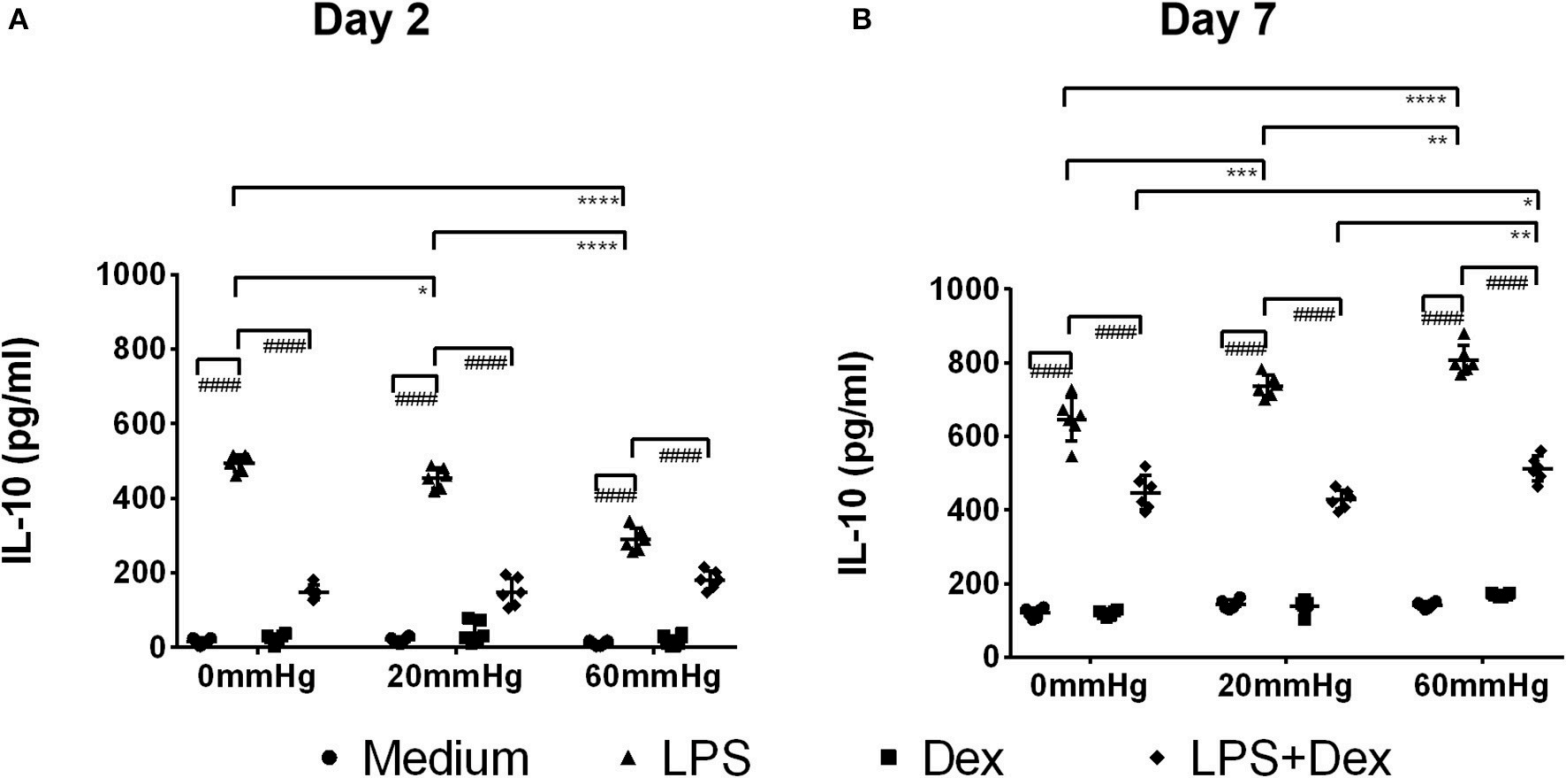
IL-10 levels in the supernatant of BMDMs under different hydrostatic pressures. Graphs displaying the calculated IL-10 levels in the supernatant from BMDMs on day 2 **(A)** and day 7 **(B)** (*n* = 6) as measured by ELISA. Statistical significance between different pressures: *p < 0.05, **p < 0.01, ***p < 0.001, ****p < 0.0001; Statistical significance between different treatments within one pressure: ^*####*^*p* < 0.0001. Data were analyzed by two-way ANOVA and Tukey's *post hoc* test.

The IL-10 expression in BMDMs on day 2 was low as determined by fluorescence microscopy and flow cytometry and no significant difference was found between the different pressures in the medium and LPS groups (Figures 6A,B, 7A,B). Meanwhile, IL-10 release into the supernatant after 2 days by LPS stimulation was statistically significant as compared to the medium group (*p* < 0.0001) (Figure 8A). IL-10 levels after LPS treatment as measured by ELISA was significantly decreased under increased HP (60 mmHg) as compared to 0 mmHg (*p* < 0.0001) or 20 mmHg (*p* < 0.0001) (Figure 8A). The difference between 0 and 20 mmHg group also reached the level of significance (*p* = 0.0161).

With Dex treatment for 2 days, as determined by immunofluorescence microscopy, the expression of IL-10 at 60 mmHg was significantly increased compared to 0 mmHg (*p* = 0.0149) or 20 mmHg (*p* < 0.0323) (Figure 6B). Similarly, flow cytometry results showed that the number of IL-10+/F4/80+ cells was higher in the 60 mmHg group than in the 0 mmHg (*p* = 0.0404) or 20 mmHg group (*p* = 0.0067) (Figure 7B). Very low IL-10 levels were detected in the supernatant of the Dex group and no significant difference could be found between the various pressures (Figure 8A). In the LPS + Dex group, no significant difference in the production of IL-10 was shown between different pressures (Figure 8A).

On day 7, no significant difference in IL-10 expression in BMDMs was found between different HPs in medium (Figure 6C). The BMDMs cultured with LPS under higher pressure (60 mmHg) on day 7 showed increased production of IL-10 as compared to 20 mmHg (*p* = 0.0100) or 0 mmHg (*p* < 0.0001). The IL-10 level at 20 mmHg was also significantly higher than at 0 mmHg (*p* = 0.0005).

The expression of IL-10 in Dex-treated BMDMs was significantly increased under higher HP (60 mmHg) compared to 0 mmHg (*p* = 0.0136) and 20 mmHg (*p* = 0.0089) as determined by fluorescence microscopy (Figure 6C).

Furthermore, flow cytometry of BMDM showed that the frequency of IL-10+/F4/80+ events at 60 mmHg was significantly higher than that in the 0 mmHg group (*p* = 0.0244) (Figure 7C).

After LPS + Dex treatment, the differences in the IL-10 expression in BMDMs as determined by fluorescence microscopy and the frequency of IL-10+/F4/80+ events under different pressure levels as determined by flow cytometry did not reach the level of significance (Figures 6, 7). BMDMs cultured with LPS+Dex under increased HP (60 mmHg) for 7 days showed higher levels of IL-10 in the supernatants than under 0 mmHg (*p* = 0.0275) or 20 mmHg (*p* = 0.0017) (Figure 8B).

### Influence of Increased Hydrostatic Pressure on the Expression of Fibronectin and Collagen IV

It has been documented that M2 macrophages, but also TM cells, produce large amounts of ECM upon culture with Dex (31, 32). Therefore, we analyzed the expression of fibronectin and collagen IV, ECM-proteins which can also be found in the TM of glaucomatous eyes, in macrophages in the cell culture setup with or without increased HP.

**Figure 9:**
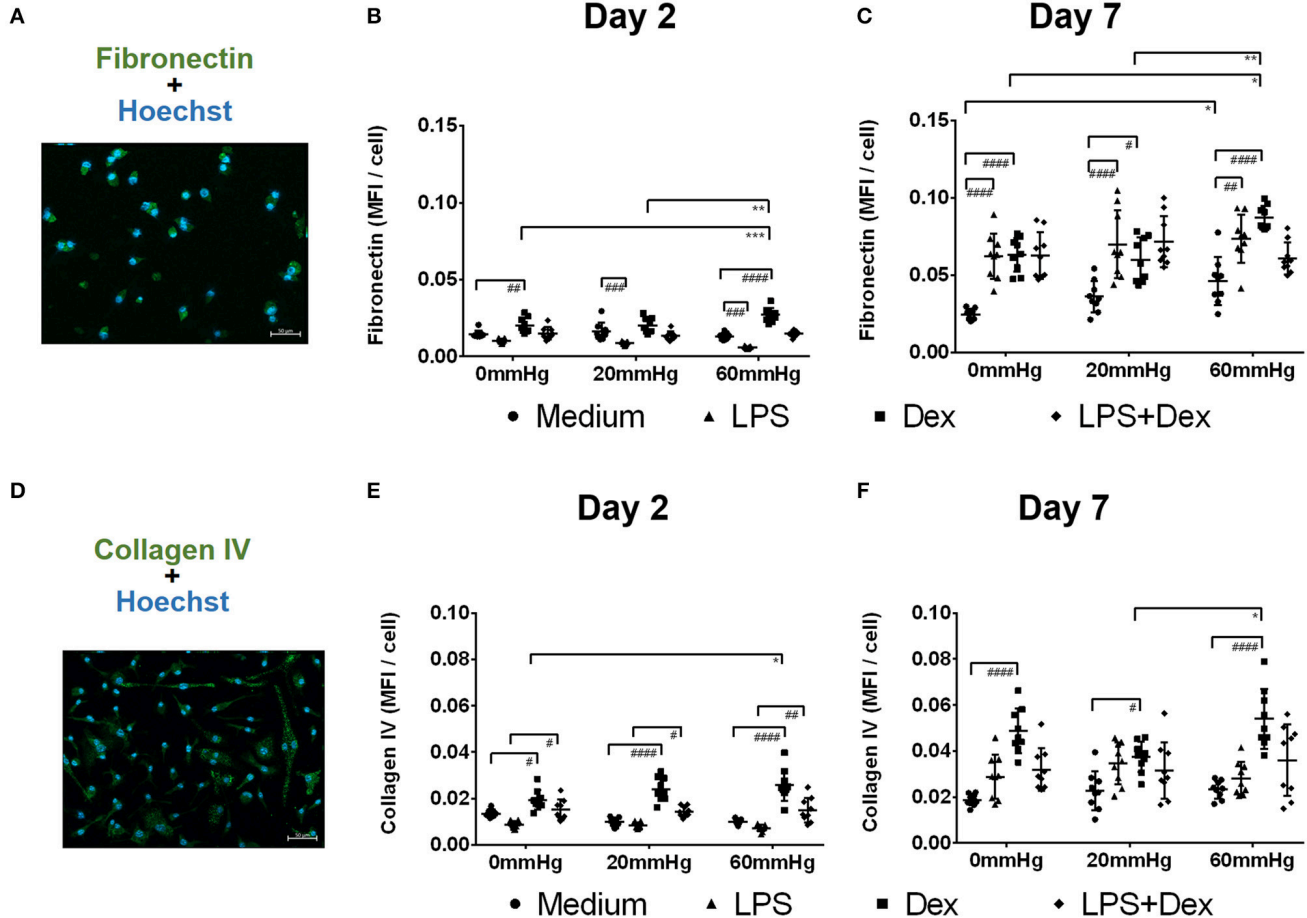
Fibronectin and collagen IV expression by BMDMs under different hydrostatic pressures. Representative immunofluorescence images of fibronectin (**A**, green) or collagen IV (**D**, green) staining in BMDMs under different HP on day 2 and day 7. Scale bar = 50 μm. Graphs displaying the calculated MFI/cell of fibronectin on day 2 **(B)** and day 7 **(C)**, and MFI/cell of collagen IV on day 2 **(E)** and day 7 **(F)** (*n* = 9). Statistical significance between different pressures was reached (*p < 0.05, **p < 0.01, ***p < 0.001); Statistical significance between different treatments within one pressure (^#^*p* < 0.05, ^*##*^*p* < 0.01, ^*###*^*p* < 0.001, ^*####*^*p* < 0.0001). Data were analyzed by two-way ANOVA and Tukey's *post hoc* test.

After 2 days, fibronectin expression was lower in LPS-stimulated BMDMs than in medium control BMDMs under the same HP level (*p* < 0.001). However, no significant difference in the fibronectin or collagen IV staining pattern was found between the various pressures in the medium or LPS group (Figures 9A,B,D,E).

After treatment of BMDMs with Dex, significantly stronger staining patterns of fibronectin (Figure 9B) and collagen IV (Figure 9E) were found on day 2 than in the medium groups (*p* < 0.05). The expression of both fibronectin and collagen IV was further increased after culture under increased HP (60 mmHg) compared to 0 mmHg (for fibronectin, *p* = 0.0009; for collagen IV, *p* = 0.0168) (Figures 9B,E). The expression of fibronectin in the 60 mmHg group was also significantly increased compared to the 20 mmHg group (*p* = 0.0011) (Figure 9B). After treatment with LPS + Dex, collagen IV staining pattern on day 2 was stronger than in the LPS group under the same pressure level (*p* < 0.05); however, no significant difference was found between various pressures in the LPS + Dex group (Figure 9E).

After prolonged culture for 7 days, BMDMs cultured at 60 mmHg with medium showed increased expression of fibronectin compared to 0 mmHg (*p* = 0.0365) (Figure 9C). On day 7, LPS-stimulated BMDMs showed significantly increased expression of fibronectin compared to medium group under the same pressure level (*p* < 0.01) (Figure 9C). No significant difference in the fibronectin or collagen IV staining pattern was found in the LPS groups between various pressures (Figures 9C,F).

Dex-treated BMDMs on day 7 showed significantly enhanced fibronectin and collagen IV staining patterns as compared to the medium group (*p* < 0.05) (Figures 9C,F). Furthermore, increased fibronectin and collagen IV expression was found for 60 mmHg as compared to 20 mmHg (fibronectin, *p* = 0.0025; collagen IV, *p* = 0.0190) (Figures 9C,F). The expression of fibronectin at 60 mmHg was higher than at 0 mmHg (*p* = 0.0122). No significant difference for either staining was found in LPS+Dex group (Figure 9C).

## Discussion

The present study investigates the influence of increased HP and the corticosteroid Dex on the M1/M2 phenotype of murine macrophages. Increased HP induced the production of the proinflammatory cytokine TNF-α and generated an M1 phenotype in macrophages after 2 days of incubation. In contrast, a prolonged exposure (7 days) under increased HP induced a pronounced M2 phenotype in these macrophages. This was associated with an increase in IL-10 and fibronectin production on day 7.

The additional treatment of macrophages with Dex for 2 days induced the production of the anti-inflammatory cytokine IL-10, the development of an M2 phenotype, and increased ECM production. Further incubation with increased HP enhanced this phenotype, as indicated by elevated IL-10 production, enhanced M2 markers, and further increased production of ECM.

Previous studies using the same pressure chamber cell culture system did not report any alteration in cell viability in the retinal photoreceptor-derived cell line (661 W) under different pressures (0, 20, or 40 mmHg) after incubation for 3 days (25). In the present study, increased HP in general induced an increase in MTT conversion in BMDM, suggesting that viability is increased. Previous reports have shown that TNF-α, which was found to be elevated under increased HP and LPS stimulation, can activate nuclear factor (NF)-κB to protect cells from apoptosis (33), which might explain the increased viability of BMDMs under increased HP. The decreased viability in Dex-treated cells may be related to the immunosuppressive potency of Dex (34).

M1 macrophages were characterized by the highly expressed surface markers CD68, CD80, CD86, major histocompatibility complex-II, and CC chemokine receptor 2 (CCR2), while M2 macrophages were characterized by the surface molecules CD163, CD206, CX3CR1, and YM1/2 (35, 36). The results of this study indicate that LPS-stimulated macrophages have a stronger M1 polarization under elevated pressure after 2 days. After 7 days, macrophages cultured under elevated pressure in the medium group polarized to M2. At the same time, the M1 population in the LPS group decreased under higher pressure.

M1/M2 can be also classified according to differences in cytokines and chemokine expression (36). Proinflammatory cytokines such as TNF-α, IL-1β, -6, -12, -18, and -23 are produced by M1 macrophages, while TGF-β1 and IL-10 are produced by M2 macrophages (37).

After culture under higher HP with LPS treatment for 2 days, numbers of TNF-α+ macrophages and levels of TNF-α release were higher, but IL-10 production was weaker. These results support the assumption that macrophages are polarized into an M1 phenotype upon higher pressure after 2 days.

Macrophage polarization into M1 phenotype under elevated pressure has been found in arterial hypertension. More CD161a+/CD68+ macrophages (both markers for M1 polarization) under higher pressure conditions were reported in spontaneously hypertensive rats (38). Inflammatory responses by M1 macrophages were thought to be responsible for premature hypertension (38). Other studies reported that by inhibiting M1 activation using Fenretinide, an anticancer agent that can perform anti-invasive and anti-metastatic functions, hypertension can be controlled in rat by activating the peroxisome proliferator-activated receptor gamma pathway (39).

After prolonged culture under higher pressure (60 mmHg) and LPS treatment for 7 days, the expression of TNF-α on day 7 was lower than on day 2 and did not show significant differences compared to macrophages under 0 or 20 mmHg. At the same time the level of IL-10 was increased when macrophages were cultured under higher pressure. This is in line with a more frequent M2 polarization on day 7, as M2 macrophages can highly express IL-10 (36, 40). In general, these findings indicated a shift in the macrophage population toward an M2 phenotype upon increased pressure after 7 days.

Our results showed notable differences examining IL-10. Dex induced IL-10 in immunofluorescence microscopy and flow cytometry, whereas this could not be shown in ELISA. Here Dex inhibited the LPS-induced IL-10 production in the supernatant. Previous studies have shown that Dex can trigger a tolerogenic stage in macrophages and dendritic cells, in which immune reactions are more likely to be inhibited (41, 42). However, another study showed that dexamethasone inhibited LPS induced IL-10 production but does not induce tolerogenic effects (43).

When using the individual methods, macrophages must be treated differently, e.g., in immunofluorescence microscopy, macrophages are cultivated on glass slides to which they do not adhere well. For the ELISA, the cells are placed on plastic plates to which they adhere very well. Such differences could be partly responsible for the difference in the observed IL-10 differences.

A previous study reported that elevated IOP induced acute proinflammatory responses, characterized by an infiltration of immune cells and upregulation of various proinflammatory cytokines, followed by proliferation and angiogenetic and fibrotic processes, associated with increased AH outflow resistance (44). A switch to macrophages with an M2 phenotype with increased CD206 and Arg-1 expression at 7–14 days after angiotensin II infusion has been described in a mouse model of hypertension, which was associated with elevated blood pressure and fibrosis (45).

In ischemia reperfusion injury (IRI), infiltration of immune cells is closely associated with inflammation and toxic effects in tissues (35). In macrophages, the activation of the TLR-4 signaling pathway induces an M1 polarization and inflammatory response that leads to activation of NF-κB to produce proinflammatory cytokines (IL-1β, TNF-α, iNOS, and others), which are known to play an important role in IRI, hemorrhagic shock, and retinal injury under elevated pressure (25, 46). Various studies reported that increased expression of TNF-α seems to be related to impaired retina in cases of acutely elevated IOP (47, 48). Except for the damage to retinal ganglion cells and optic nerve head, the elevated production of TNF-α by M1 macrophages induces a response of T-helper cells and their differentiation to Th17, which turns the acute inflammation into a fibrotic reaction (49). In retinal IRI, inflammatory reaction normally starts after a few hours and lasts several days (generally 7 days), while the chronic phase starts after a few days and lasts for months (48). M2 macrophages seem to be responsible for repair processes in the chronic phase (50). IL-10 has been reported to be upregulated 144 h after starting the IRI model, and high levels of TNF-α still also could be detected at this point in time (48). A similar M1 to M2 shifting was also reported in oxygen-induced retinopathy in mice (51).

Not only can Dex treatment suppress the proinflammatory response, Dex may also induce macrophages to differentiate into an alternatively activated phenotype (M2) (52). This is in line with the present study, where BMDMs exposed to Dex switched to an M2 polarization, even when macrophages were treated with LPS. In our view, GC inhibits the activation of M1 macrophages and may induce anti-inflammatory macrophages, which agrees with other recent reports (53). This finding is also in line with a previous study in mice, showing that Dex treatment inhibited LPS-induced TNF-α and IL-6 gene expression in macrophages (54). The same study demonstrated that after genetic depletion of the GC receptor, macrophages produced increased levels of TNF-α and IL-6 in response to LPS stimulation.

We investigated fibronectin and collagen IV in macrophages, because they play an important role in regulating intraocular pressure in the eye and are also involved in the development of glaucoma (55). They occur in the basement membrane of the trabecular meshwork and are produced by local cells. They are regulated by transforming growth factor-β2, which can be found in aqueous humor. Glucocorticoids can induce transforming growth factor-β2 (56), and thus cause an increased IOP (57, 58) and increased expression of fibronectin synthesis (55, 59).

In the present study, BMDMs in medium or LPS groups cultured under increased HP for 2 days showed less fibronectin and collagen IV production. However, on day 7 the production of fibronectin and collagen IV was increased in LPS as compared to medium. Previous studies reported that macrophages at wound sites in a mice model switch from M1 to M2 with an increased wound healing profile from days 5 to 10 (60). M2 macrophages can produce large amounts of arginase-1 and TGF-β1, and thereby contribute to the fibrotic process (45). Fibrosis also seems to be closely related to GC-induced ocular hypertension (61). In the eye, GC treatment can lead to more ECM deposition in the outflow pathway and obstruct outflow (62, 63). After treatment with Dex, BMDMs displayed more ECM (fibronectin and collagen IV) production after culture under higher HP for 2 or 7 days. It has been shown previously that GCs can block the LPS or mechanical stress-mediated proinflammatory responses in macrophages (64). Suppressed proinflammatory responses have been found to be related to impaired ECM turnover (65). The increased M2 polarization of GC-exposed macrophages in the outflow pathway owing to higher pressure may be related to the pathogenesis of GC-induced ocular hypertension or glaucoma.

IOP may become elevated in uveitis patients and some ultimately develop uveitis glaucoma. The majority of uveitis glaucoma patients suffer from secondary open-angle glaucoma while only few of them have angle closure (66). Trabeculitis and clogging of the TM with inflammatory cells (most commonly seen in viral uveitis) may lead to a reduced AH outflow and acute IOP elevation with open angles (67). The chronic changes in the TM outflow pathway (ECM deposition, changing the structure or function in TM cells) occurring during inflammatory activity (68) and corticosteroid treatment are thought to be responsible for uveitis glaucoma (69). The change in the phenotype of macrophages and the underlying function, as presented herein, may play an important role in the development and deterioration of uveitis glaucoma. We suspect that some macrophages remain in the eye after uveitis. After inflammation in the eye, the remaining macrophages probably already show an M2 phenotype, which tends to promote fibrotic mechanisms and can therefore also be involved in increasing IOP. Further treatment of the eye with Dex or an increase in IOP would likely increase this phenotype and accelerate the development of secondary glaucoma.

In summary, we compared the functional capacity of BMDMs under different HPs with respect to M1 and M2 phenotypes and effector functions. Increased HP provokes a time-dependent primary M1-related immune response followed by a secondary M2 response. Dex treatment of macrophages and increased HP showed synergistic effects, reflected by a pronounced M2 phenotype induction. The results imply that increased HP may affect the outcome of inflammation and measures extenuating such a mechanism could help to manage secondary glaucoma more effectively.

## Data Availability Statement

All datasets presented in this study are included in the article/Supplementary Material.

## Ethics Statement

The protocol was approved by the North Rhine-Westphalia State Agency for Nature, Environment, and Consumer Protection (LANUV) (AZ 81-02.05.50.19.006). The use of animals was in accordance with the Institutional Animals Care and Use and Ethics Committee, and with the ARVO Statement for the Use of Animals in Ophthalmic and Vision Research.

## Author Contributions

BW, DB, and CH designed the study and wrote the manuscript. BW, MK, BL, GM, SW, MB, TJ, ST, AH, DB, and CH performed the experiments and analyzed the data. All authors contributed to the article and approved the submitted version.

## Conflict of Interest

The authors declare that the research was conducted in the absence of any commercial or financial relationships that could be construed as a potential conflict of interest.

## Supplementary Material

Supplementary Figure 1 Identification of primary bone marrow-derived cells. Whole events from one sample on day 7 in the SSC/FCS window. Ungated events are displayed in SSC-H/SSC-A dot plot. Single live cell gate was set and positive events are depicted in the left area. After dead cells were excluded, F4/80 isotype control and F4/80-positive events are shown in the right-side gate.

## Abbreviations

BMDMs: bone marrow-derived macrophages
Dex: dexamethasone
ECM: extracellular matrix
GCs: glucocorticoids
HP: hydrostatic pressure
IOP: intraocular pressure
IRI: ischemia reperfusion damage
LPS: lipopolysaccharide
MFI: mean fluorescence intensity
SD: mean ± standard deviation
OD: optical density
TLR-4: Toll like receptor-4
TM: trabecular meshwork.
