# Supplementary figures and images for: Increased Hydrostatic Pressure Promotes Primary M1 Reaction and Secondary M2 Polarization in Macrophages
Source: Front Immunol. 2020 Oct 14;11:573955. doi: 10.3389/fimmu.2020.573955 (PMC7591771; doi:10.3389/fimmu.2020.573955)

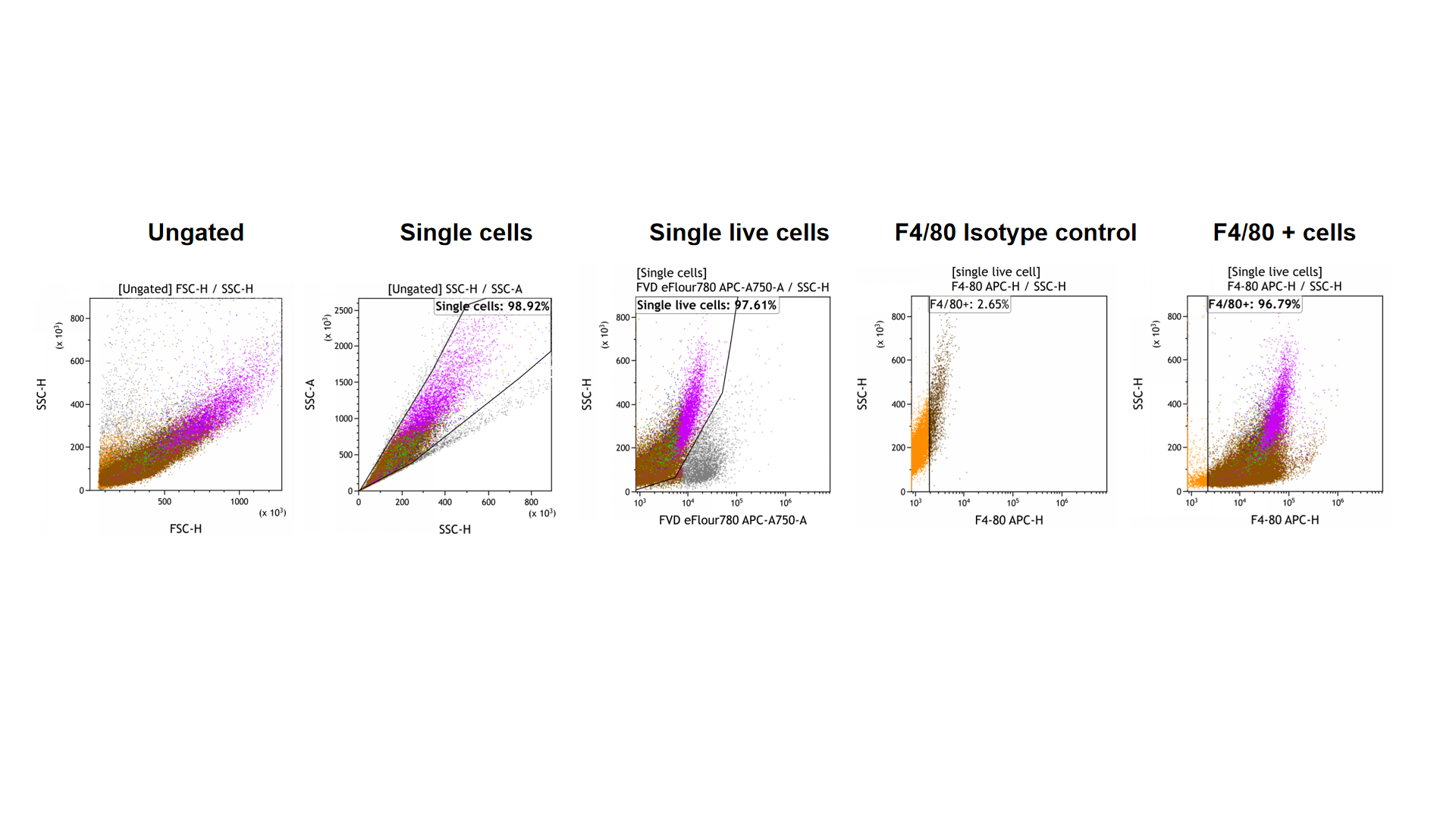

Supplement: Supplementary Figure 1 — Identification of primary bone marrow-derived cells. Whole events from one sample on day 7 in the SSC/FCS window. Ungated events are displayed in SSC-H/SSC-A dot plot. Single live cell gate was set and positive events are depicted in the left area. After dead cells were excluded, F4/80 isotype control and F4/80-positive events are shown in the right-side gate. [file Image_1.tif]
